# Herpes simplex encephalitis following ChAdOx1 nCoV-19 vaccination: a case report and review of the literature

**DOI:** 10.1186/s12879-022-07186-9

**Published:** 2022-03-03

**Authors:** Mohammadreza Moslemi, Mohammadreza Ardalan, Morteza Haramshahi, Homa Mirzaei, Sahba Khosousi Sani, Ramtin Dastgir, Nima Dastgir

**Affiliations:** 1 Department of Internal Medicine, School of Medicine, Tabriz University of Medical Sciences, Tabriz, Iran; 2 Kidney Research Center, Tabriz University of Medical Sciences, Tabriz, Iran; 3 Department of Oral Medicine, School of Dentistry, Shahid Beheshti University of Medical Sciences, Tehran, Iran; 4 Faculty of Dentistry, Tehran Medical Sciences, Islamic Azad University, Number 9, Neyestan 9 Avenue, Pasdaran Street, Tehran, Iran; 5 Department of Infectious Diseases, Imam Ali Hospital, Hormozgan University of Medical Sciences, Roudan, Iran

**Keywords:** COVID-19, SARS-CoV-2, COVID-19 vaccines, Herpes simplex encephalitis, Herpes simplex, Case report

## Abstract

**Background:**

Ever since the administration of early doses of COVID-19 vaccines, instances of adverse effects have been reported. Viral infections, specifically herpes simplex reinfection and coinfections, have been reported following administration of different types of vaccines. To our knowledge, there have not been any reports of herpes simplex encephalitis following administration of any type of COVID-19 vaccine to date.

**Case presentation:**

In this article intends to report a case of herpes simplex encephalitis in a 27-year-old male patient who was vaccinated with the ChAdOx1 nCoV-19 vaccine.

**Conclusions:**

Our study suggests a possible but very rare side effect of ChAdOx1 nCoV-19 vaccine, which requires immediate medical attention and can lead to devastating consequences if left undiagnosed and untreated.

## Background

Ever since the outbreak of COVID-19 pandemic, development of effective and safe vaccines against severe acute respiratory syndrome coronavirus 2 (SARS-CoV-2) has been a crucial concern to the scientific community of the world in order to tackle this global emergency. The first mass vaccination program started back in early December 2020, and the number of total vaccination doses administered worldwide has surpassed 6.5 billion doses as of November 1st, 2021 [1]. There have been at least 13 different vaccines (across four platforms) administered throughout this time [2]. ChAdOx1 nCoV-19 (codenamed AZD1222) was developed at Oxford University and consists of a replication-deficient chimpanzee adenoviral vector ChAdOx1, containing the SARS-CoV-2 structural surface glycoprotein antigen (spike protein; nCoV-19) gene and has been one of the widely accepted and administered vaccines worldwide [3]. However, since the administration of early doses of COVID-19 vaccines, instances of adverse effects have been reported all over the world. Viral infections, specifically herpes simplex and cytomegalovirus reinfection and coinfections have been reported following administration of different types of vaccines [4–7]. Furthermore, herpes virus reactivation has been previously reported following administration of Hepatitis A, rabies, and trivalent influenza vaccines which can be suggestive of a vaccine-modulated immunomodulation process [8].

To our knowledge, there have not been any reports of herpes simplex encephalitis following administration of any type of COVID-19 vaccine to date and we report the first case. In this article, we intend to report a case of herpes simplex encephalitis in a 27-year-old male patient who was vaccinated with the ChAdOx1 nCoV-19 vaccine.

## Case presentation

We intend to report a case of a 27-year-old male patient with no underlying systemic conditions and no prior history of herpes simplex virus (HSV) and COVID-19 infection who received his first dose of Oxford–AstraZeneca (ChAdOx1 nCoV-19) COVID-19 vaccine (codenamed AZD1222) 8 days prior to being referred to us. The first couple of days after the administration of the vaccine were uneventful. Three days after the administration of the vaccine, the patient woke up and immediately started vomiting. Following the patient’s emesis period, symptoms of severe headache and altered mental status began to appear, including slowed psychomotor activity and loss of alertness; however, no signs of disorientation to time and location and people around him were evident at that time. The patient then presented to a local clinic for outpatient treatment and was prescribed baclofen and diclofenac. After returning home, the patient then developed symptoms of severe headache, agitation, delirium, and disorientation to time, location, and people around him. The same night patient experienced severe emetic episodes again. The next morning, following four days after administering the first dose of vaccine, he presented to the emergency department of a local hospital. After obtaining blood tests, chest computed tomography (CT) scan, and brain CT scan, he was transferred to Imam Reza hospital (Tabriz, Iran) for further evaluations with the preliminary diagnosis of encephalitis. Chest and brain CT scans yielded normal findings with no evidence of involvement. Our primary neurological evaluations revealed altered mental status to Glasgow Coma Scale (GCS) score of 12 [eye opening only to verbal commands (3), normal motor response (6), and inappropriate words on verbal commands (3)]. Neurological examination revealed altered mental status and decreased level of consciousness, and disorientation. Furthermore, there were no evidences of ataxia, hemiparesis, and aphasia. There were no focal neurological deficits observed in the primary evaluations.

Following these observations, the decision was made to intubate the patient in order to preserve the airway, due to reduced levels of consciousness and admit him to the intensive care unit. Results obtained from complimentary evaluations revealed elevated temperature of 38.9 °C (102.02 °F) and leukocytosis (12,900/mm^3^). The remaining laboratory test values were all in normal ranges. The patient was then immediately put on Acyclovir 10 mg/kg TDS due to suspicion of herpetic encephalitis based on his clinical presentations and medical history. Cerebrospinal fluid (CSF) analysis demonstrated decreased protein levels (3.05 mg/dl), a WBC count of 600 per mm^3^ with the predominance of lymphocyte, and normal glucose levels, all of which are suggestive of a viral encephalitis.

Polymerase chain reaction (PCR) test showed positive for HSV in cerebrospinal fluid, and therefore, the diagnosis of herpes simplex encephalitis was established. Furthermore, it is established that the identification of the viral DNA in the cerebrospinal fluid via PCR is the most sensitive and specific for the diagnosis of HSV encephalitis [16]. Moreover, brain magnetic resonance imaging (MRI) was performed using axial T1, T2, and sagittal T2/W images, which revealed no space-occupying lesions in supra- and infratemporal structures. White and gray matter signals, cerebral ventricles, major intracranial vascular structures, basal ganglia, and brainstem were all normal. Furthermore, electroencephalography (EEG) also depicted normal results.

The patient was planned for 21 days of Acyclovir treatment. His altered mental status gradually ameliorated over the course of the first eight days of admission and was extubated after the 8th day. Although some degrees of amnesia and unusual behaviors were still present following the extubation, these symptoms improved over the next few days, and the patient was finally discharged on the 21st day.

## Discussion and conclusion

Encephalitis is defined as inflammation of the brain parenchyma with neurologic dysfunction, resulting from infectious, postinfectious, and noninfectious etiologies. Infectious etiologies constitute roughly 50% of all cases and are the most diagnosed etiologic factor of encephalitis [9, 10]. HSV is the most common cause of viral encephalitis and should raise suspicion in patients who present with sudden alteration in levels of consciousness that otherwise remain unexplained. HSV-1 is one of eight identified human herpesviruses. The primary route through which HSV enters a host are impaired or breached mucous and dermal membranes. After the primary infection of the dermal or mucosal epithelial layers, the HSV then infects sensory neurons via interactions with glycosaminoglycans available on cell-surfaces [11, 12]. The mechanisms by which HSV penetrates the central nervous system (CNS) still remains unclear. The most probable routes by which HSV accesses CNS include retrograde transportation through the trigeminal (CN V) or olfactory (CN I) nerves or via hematogenous dissemination [13–15]. HSV-1 is responsible for the great majority of herpes simplex encephalitis cases; however, it has been recently shown that HSV-2 infections can also present with encephalitis [16].

Ever since the early days of COVID-19 pandemic breakout, cases of herpes simplex or herpes zoster-like coinfections have been reported [4, 17]. This can be further clarified via the function of natural killer group 2D (NKG2D) ligands, also known as “stress-induced ligands,” which can be found on normal, healthy cell surfaces such as neurons as they act to avoid auto-reactivity of natural killer T cells against normal, healthy host tissues. HSV and varicella-zoster virus (VZV) result in downregulation of NKG2D ligands on neuron cell membranes and neural terminals after infecting them. Therefore, these neurons cannot be detected by natural killer cells, consequently HSV or VZV infections enter their latent phase [18, 19]. This downregulation results in a balance between natural killer cells activation and viral evasion and results in viral latency, hence disruption of this balance can impact the outcome of herpetic infections and possible reactivation [31]. The above-mentioned ligands are upregulated on the cell membranes following numerous stress-inducing situations, including oncogenic activation, hypoxia, and viral infections. The two latter conditions are seen in COVID-19 infections, which may describe the coincidence of COVID-19 coinfection with HSV or VZV by disrupting the aforementioned balance [20, 21, 31].

Furthermore, ChAdOx1 nCoV-19 vaccine is a replication-deficient simian adenoviral vector which expresses the full-length SARS-CoV-2 spike protein. It has been demonstrated that this vaccine can be initiative of cytokine release and an immune response cascades in a similar fashion to viral infections of SARS-CoV-2 itself. This mechanism can further lead to the upregulation of NKG2D ligands and reactivation of the HSV from its latent phase as described above and development of the clinical signs and symptoms of herpetic infections [22]. This mechanism can explain numerous herpes zoster cutaneous coinfections following administration of different types of COVID-19 vaccine [4–6, 23–29]; however, there are currently no other reports of herpes simplex encephalitis following COVID-19 vaccination, though there has been a single report of encephalitis following administration of ChAdOx1 nCoV-19 vaccine in which the diagnosis of herpetic sources was ruled out [30].

Our study suggests a possible but very rare side effect of ChAdOx1 nCoV-19 vaccine, which requires immediate medical attention and can lead to devastating consequences if left undiagnosed and untreated. However, due to scarcity of similar evidence, reaching a definitive conclusion regarding the correlation of the ChAdOx1 nCoV-19 vaccine and herpes simplex encephalitis is unattainable.

## Data Availability

Not applicable.

## Abbreviations

SARS-CoV-2: Severe acute respiratory syndrome coronavirus 2
CT: Computed tomography
CSF: Cerebrospinal fluid
PCR: Polymerase chain reaction
MRI: Magnetic resonance imaging
EEG: Electroencephalography
HSV: Herpes simplex virus.
HHV: Human herpesviruses
VZV: Varicella-zoster virus
CNS: Central nervous system
NKG2D: Natural killer group 2D
